# *Helicobacter pylori* antibody and pepsinogen testing for predicting gastric microbiome abundance

**DOI:** 10.1371/journal.pone.0225961

**Published:** 2019-12-04

**Authors:** Saemi Choi, Jae Gon Lee, A-reum Lee, Chang Soo Eun, Dong Soo Han, Chan Hyuk Park

**Affiliations:** 1 Department of Medicine, Hanyang University College of Medicine, Seoul, Korea; 2 Department of Internal Medicine, Hanyang University Guri Hospital, Hanyang University College of Medicine, Guri, Korea; Oita University Faculty of Medicine, JAPAN

## Abstract

**Background:**

Although the high-throughput sequencing technique is useful for evaluating gastric microbiome, it is difficult to use clinically. We aimed to develop a predictive model for gastric microbiome based on serologic testing.

**Methods:**

This study was designed to analyze sequencing data obtained from the Hanyang University Gastric Microbiome Cohort, which was established initially to investigate gastric microbial composition according to the intragastric environment. We evaluated the relationship between the relative abundance of potential gastric cancer-associated bacteria (nitrosating/nitrate-reducing bacteria or type IV secretion system [T4SS] protein gene-contributing bacteria) and serologic markers (IgG anti-*Helicobacter pylori* [HP] antibody or pepsinogen [PG] levels).

**Results:**

We included 57 and 26 participants without and with HP infection, respectively. The relative abundance of nitrosating/nitrate-reducing bacteria was 4.9% and 3.6% in the HP-negative and HP-positive groups, respectively, while that of T4SS protein gene-contributing bacteria was 20.5% and 6.5% in the HP-negative and HP-positive groups, respectively. The relative abundance of both nitrosating/nitrate-reducing bacteria and T4SS protein gene-contributing bacteria increased exponentially as PG levels decreased. Advanced age (only for nitrosating/nitrate-reducing bacteria), a negative result of IgG anti-HP antibody, low PG levels, and high Charlson comorbidity index were associated with a high relative abundance of nitrosating/nitrate-reducing bacteria and T4SS protein gene-contributing bacteria. The adjusted coefficient of determination (R^2^) was 53.7% and 70.0% in the model for nitrosating/nitrate-reducing bacteria and T4SS protein gene-contributing bacteria, respectively.

**Conclusion:**

Not only the negative results of IgG anti-HP antibody but also low PG levels were associated with a high abundance of nitrosating/nitrate-reducing bacteria and T4SS protein gene-contributing bacteria.

## Introduction

Gastric cancer is one of the leading health problems worldwide, accounting for an estimated 990,000 deaths yearly, making it the third and fifth leading causes of cancer-related deaths in men and women, respectively [1]. Various factors including age, male sex, tobacco smoking, family history of gastric cancer, high intake of smoked and salty foods, small intake of vegetables and fruits, and low socioeconomic status have been known to be associated with the development of gastric cancer [2]. However, *Helicobacter pylori* (HP) infection is the most potent known risk factor for gastric cancer, and over 50% of the global population over 40 years old has HP colonization in the stomach [3].

Efforts have been made to prevent gastric cancer development by eradicating HP; however, a previous randomized controlled trial showed that successful eradication does not entirely guarantee the prevention of gastric cancer [4]. Additionally, HP often disappears spontaneously in elderly patients with the progression of atrophic gastritis and intestinal metaplasia, which are precancerous lesions of gastric cancer [5,6]. In addition to HP, many factors affect the development of gastric cancer, such as bacterial overgrowth, nitrate reduction, and *N*-nitroso carcinogens [7]. Previous studies suggest that various bacteria including *Clostridium*, *Staphylococcus*, and *Neisseria* may play a role in the formation of *N*-nitroso compounds, which increase the risk of gastric cancer [8–11]. In addition, we demonstrated that metagenomes derived from intragastric bacteria other than HP differed according to the intragastric environment–gastritis, intestinal metaplasia, and cancer [12]. The type IV secretion system (T4SS) protein genes, which are essential for transferring cytotoxin-associated gene A (CagA) from HP into the human gastric epithelium, were abundant in the stomach of patients with intestinal metaplasia [12].

Therefore, we should also focus on gastric microbiome other than HP in understanding gastric carcinogenesis and assessing the individual risk of gastric cancer. During the past decade, the high-throughput sequencing technique known as next-generation sequencing has been introduced for the analysis of environmental microbiome [13]. Using this technique, we discovered that the composition of intragastric microbiome has characteristics that differ according to the disease status [13,14]. However, gastric microbiome analysis using next-generation sequencing may be ineffective for risk stratification because it requires a gastric mucosal biopsy or gastric juice sample and the sequence data is relatively difficult to analyze. In contrast, serologic testing including IgG anti-HP antibody and pepsinogen (PG) is less invasive and easy to interpret the results compared to microbiome analysis using next-generation sequencing. PG I is produced by the chief and mucous neck cells in the fundic glands, while PG II is produced by these cells and the pyloric and Brunner’s glandular cells [15]. It is widely accepted that the serum PG levels reflect the functional and morphologic status of the gastric mucosa [15]. Therefore, we hypothesized that the composition of gastric microbiome could be predicted using serologic testing of IgG anti-HP antibody and PG levels. Consequently, in this study, we aimed to develop a predictive model for gastric microbiome detection using serologic testing.

## Methods

### Study design

This study was designed to analyze the sequence data obtained from the Hanyang University Gastric Microbiome Cohort, which was established initially to investigate gastric microbial composition according to the intragastric environment (KCT0001602, https://cris.nih.go.kr). The detailed results of gastric microbiome analysis were published in the previous study [12]. Written informed consent was obtained from all participants. The study protocol conforms to the ethical guidelines of the 1975 Declaration of Helsinki, as reflected in a priori approval by the institution’s human research committee. This study was approved by the Institutional Review Board on Human Subjects Research and Ethics Committee, Hanyang University Guri Hospital, Korea on April 26, 2018 (GURI 2018-04-029).

Hanyang University Gastric Microbiome Cohort consisted of healthy volunteers or patients with dyspepsia who were scheduled for esophagogastroduodenoscopy. All participants in the cohort underwent endoscopic biopsy for gastric microbiome analysis and serologic testing of IgG anti-HP antibody and PG. Demographic information of patients including age, sex, weight, height, smoking habits, and comorbidities was also collected. Screened patients who met the following criteria were excluded from participation in the cohort: (a) patients who were diagnosed with gastric cancer by endoscopy with biopsy; (b) patients who took acid-suppressive agents including proton pump inhibitors and H_2_ receptor antagonists, mucoprotective agents, probiotics, or antibiotics, within 3 months prior to enrollment; (c) patients with a history of gastric neoplastic lesions and (d) patients who underwent a gastrectomy.

### Sample acquisition and serologic testing

For analysis of microbiome composition, we obtained four pieces of the gastric mucosal tissue through an endoscopic biopsy at the greater curvature side of the mid-antrum. HP infection status was evaluated by a rapid urease test. For serologic assessments, serum IgG anti-HP antibody and PG I/II testing were performed using enzyme and latex agglutination turbidimetric immunoassays, respectively.

### DNA extraction

The DNA was extracted from the gastric mucosal tissue as previously described [12,13]. Briefly, 100 mg frozen gastric mucosal tissues was suspended in 750 μL sterile bacterial lysis buffer (200 mmol/L sodium chloride [NaCl], 100 mmol/L ethylenediaminetetraacetic acid [EDTA, pH 8.0], 20 mmol/L Tris base, and 20 mg/mL lysozyme). After incubation at 37°C for 30 minutes, 20 μL proteinase K and 80 μL 10% sodium dodecyl sulfate (SDS) were added to the mixture. Then, it was incubated at 65°C for 30 minutes. Finally, bead beating was performed for 90 seconds at 6.9 g (PRECELLYS 24; Bertin Technologies, Le Bretonneux, France) following adding 300 mg of 0.1 mm zirconium beads (BioSpec Products, Bartlesville, OK, USA) to complete the homogenization. The homogenized mixture was cooled on ice, followed by centrifuging at 18.3 g for 5 minutes. DNA was extracted from the supernatant using phenol/chloroform/isoamyl alcohol (25:24:1) followed by chloroform/isoamyl alcohol (24:1). Then it was precipitated with absolute ethanol at -20°C for 1 hour. The DNA was suspended in DNase-free H_2_O and cleaned using a DNA clean-up kit (QIAGEN, Hilden, Germany). Isolated DNA was stored at -80°C until the microbial characterization.

### 16S rRNA gene sequencing and analysis of microbial composition

16S rRNA gene sequencing was performed as previously described [12]. Each sequenced sample was prepared according to the Illumina 16S Metagenomic Sequencing Library protocol. The quantification of DNA and the DNA quality assessment was done by PicoGreen and Nanodrop, respectively. Extracted DNA was amplified with the following 16S V3–V4 primers: forward, 5′-TCGTCGGCAGCGTCAGATGTGTATAAGAGACAGCCTACGGGNGGCWGCAG-3′ and reverse, 5′-GTCTCGTGGGCTCGGAGATGTGTATAAGAGACAGGACTACHVGGGTATCTAATCC-3′. Then, a subsequent limited‐cycle amplification step was performed to add multiplexing indices and Illumina sequencing adapters. The final products were normalized and pooled using PicoGreen and the size of libraries were verified using the TapeStation DNA screentape D1000 (Agilent). We then sequenced the DNA using the Illumina MiSeq system (Illumina, San Diego, CA, USA) according to the manufacturer’s instructions and the sequence data were processed using QIIME version 1.9.0 [16].

Low-quality reads contained incorrect primer sequences or more than one ambiguous base were excluded. The remaining reads were classified into the groups based on their unique nucleotide barcodes. To avoid potential bias due to copy number variation, the read count was normalized to the corresponding copy number of 16S rRNA genes [17]. Taxonomic composition and bacterial diversity for each sample were identified based on a 97% similarity with the GreenGenes database (version 13.5) using QIIME.

DNA sequences obtained from this project have been deposited in the National Center for Biotechnology Information (NCBI) short read archive under the Accession No. SRP109017.

### Relative abundance of gastric carcinogenesis-related bacteria

To quantify the composition of gastric carcinogenesis-related bacteria, we investigated the relative co-abundance of several bacteria of interest. First, we calculated the relative abundance of the nitrosating/nitrate-reducing bacteria, which are defined as bacteria with the ability to nitrosate or reduce nitrate [14]. The following bacterial taxa were included in the analysis: *Citrobacter*, *Escherichia*, *Haemophilus influenzae*, *Haemophilus parainfluenzae*, *Klebsiella*, *Neisseria*, *Pseudomonas*, *Staphylococcus aureus*, *Staphylococcus epidermidis*, *Veillonella*, and Xanthomonadaceae. Then, we analyzed the relative abundance of T4SS protein gene-contributing bacteria, which are defined as bacteria with a T4SS protein gene in their genome [12]. We included the following taxa: Xanthomonadaceae, Pseudomonadaceae, Pasteurellaceae, Legionellaceae, Enterobacteriaceae, Campylobacteriaceae, Neisseriaceae, Comamonadaceae, Burkholderiaceae, Alcaligenaceae, Sphingomonadaceae, Erythrobacteraceae, Rickettsiaceae, Rhodobacteraceae, Rhizobiales, Caulobacteraceae, Flavobacteriaceae, Solibacteraceae, Koribacteraceae, and Acidobacteriaceae.

To better understand the association between serologic markers and potential gastric carcinogenesis-related bacteria, we further investigated two other bacterial subgroups. The first was phylum Cyanobacteria, which is known to be abundant in the stomachs of individuals with no history of HP infection [12]. It is known that polysaccharides derived from Cyanobacteria prevent HP attachment to the gastric mucosa [18,19]. The second additional bacterial subgroup analyzed was the type III secretion system (T3SS) protein gene-contributing bacteria; the following taxa were included: Aeromonadaceae, Alcaligenaceae, Beijerinckiaceae, Bradyrhizobiaceae, Burkholderiaceae, Caulobacteraceae, Cloacamonaceae, Comamonadaceae, Criblamydiaceae, Desulfovibrionaceae, Enterobacteriaceae, Halomonadaceae, Hyphomicrobiaceae, Myxococcaceae, Oxalobacteraceae, Pasteurellaceae, Phyllobacteriaceae, Pseudomonadaceae, Rhizobiaceae, Sphingomonadaceae, Succinivibrionaceae, Thiotrichaceae, Verrucomicrobiaceae, Xanthomonadaceae.

### Statistical analysis

Continuous and categorical variables were expressed as means ± standard deviation and numbers (with proportions), respectively. *t*-test and chi-square test were performed to group comparisons for continuous and categorical variables, respectively. To identify the difference in the gastric microbiome composition of patients with HP infection and those without HP infection, the relative abundance of bacteria at the phylum level was analyzed. Additionally, linear discriminant analysis was performed to evaluate the predominant bacterial taxa in the HP-positive and HP-negative groups.

The relationship between bacterial abundance and PG was assessed using the scatter plot and regression line according to IgG anti-HP antibody status. To identify potential outliers, the Bonferroni outlier test was performed [20] while the multiple regression analysis was used to adjust potential confounding factors such as age, sex, and comorbidities. *P* < 0.05 was considered significant for group comparisons. Finally, we performed a permutational analysis of variance with Bray-Curtis dissimilarity based on 1,000 permutations of the data to investigate the association of various clinical factors, including HP antibody activity and PG levels, with gastric microbiome composition [21]. All statistical procedures were conducted using R (version 3.6.0; R Foundation for Statistical Computing, Vienna, Austria).

## Results

### Baseline characteristics and microbiome reads

**Table 1** shows baseline patient characteristics and microbiome reads according to HP infection status. Of the 83 included participants, 26 (31.3%) had HP infection. Although the mean age of the HP-positive group tended to be higher than that of the HP-negative group, a significant difference was not identified. In the HP-negative group, no participant showed positive IgG anti-HP antibody results, whereas 1 (1.8%) and 56 (98.2%) showed equivocal and negative IgG anti-HP antibody results, respectively. In the HP-positive group, 20 (76.9%), 3 (11.5%), and 3 (11.5%) showed positive, equivocal, and negative IgG anti-HP antibody results, respectively. Both PG I and II in the HP-positive group were higher than those in the HP-negative group [HP-negative vs. HP-positive: PG I, 52.7 ± 32.5 vs. 83.0 ± 49.4, *P* = 0.007 and PG II, 11.9 ± 9.0 vs. 28.5 ± 13.1, *P* < 0.001]. However, the PG I/II ratio in the HP-negative group was higher than that in the HP-positive group [HP-negative vs. HP-positive, 4.8 ± 1.6 vs. 2.9 ± 0.9, *P* < 0.001]. The Charlson comorbidity index did not differ between the groups (*P* = 0.413).

**Table 1:** Baseline patient characteristics and microbiome reads of samples obtained from our study.

Variable		*H*. *pylori* (-)	*H*. *pylori* (+)	*P*-value
n		57	26	
Age, year, mean±SD		37.9±16.7	45.0±18.5	0.098
Male, n (%)		27 (47.4)	12 (46.2)	0.918
IgG anti-*H*. *pylori* antibody, n (%)				<0.001
	Negative	56 (98.2)	3 (11.5)	
	Equivocal	1 (1.8)	3 (11.5)	
	Positive	0 (0.0)	20 (76.9)	
Pepsinogen testing, mean±SD				
	Pepsinogen I, ng/mL	52.7±32.5	83.0±49.4	0.007
	Pepsinogen II, ng/mL	11.9±9.0	28.5±13.1	<0.001
	Pepsinogen I/II ratio	4.8±1.6	2.9±0.9	<0.001
Body mass index, kg/m^2^, mean±SD		22.7±3.5	22.7±4.1	0.998
Smoking habit, n (%)				0.783
	Never	37 (64.9)	18 (69.2)	
	former	10 (17.5)	3 (11.5)	
	Current	10 (17.5)	5 (19.2)	
Charlson comorbidity index, n (%)				0.413
	0	53 (93.0)	23 (88.5)	
	1	3 (5.3)	2 (7.7)	
	2	0 (0.0)	1 (3.8)	
	3	1 (1.8)	0 (0.0)	
Microbiome reads, mean±SD				
	Read count	8409.3±5659.8	18977.1±6923.8	<0.001
	OTU	269.7±151.7	152.7±81.6	<0.001
	Observed species	121.8±78.7	78.7±29.5	<0.001
	Chao1 estimator	151.1±60.2	94.7±31.4	<0.001
	Shannon's diversity index	3.76±0.40	1.20±1.01	<0.001
	Simpson's diversity index	0.95±0.03	0.35±0.29	<0.001

OTU, operational taxonomic unit; SD, standard deviation

The relative abundance of bacteria at the phylum level in the HP-negative and HP-positive groups is shown in **S1 Fig**. The proportion of Firmicutes and non-HP Proteobacteria was 29.8% and 29.5%, respectively, in the HP-negative group, and 4.2% and 6.6%, respectively, in the HP-positive group. In the HP-positive group, the proportion of *Helicobacter* was 81.0%. In the linear discriminant analysis, various bacterial taxa were abundant in the HP-negative group, whereas a few bacterial taxa, including HP, were significant in the HP-positive group (**S2 Fig**).

### Relative abundance of gastric carcinogenesis-related bacteria

The relative abundance of nitrosating/nitrate-reducing bacteria and T4SS protein gene-contributing bacteria is shown in **S3 Fig**. The relative abundance of nitrosating/nitrate-reducing bacteria was 4.9% and 3.6% in the HP-negative and HP-positive groups, respectively, while *Neisseria* was the predominant bacterial taxon (1.3% and 1.4%, respectively). The relative abundance of T4SS protein gene-contributing bacteria was 20.5% and 6.5% in the HP-negative and HP-positive groups, respectively.

### Relationship between bacterial abundance and PG level

PG I and II levels according to the HP infection are shown in **S4 Fig**. Two outliers (one each for PG I and PG II) were identified using the Bonferroni outlier test, and they were excluded in the analysis of predictive model development due to avoid overfitting of the model.

**Fig 1:**
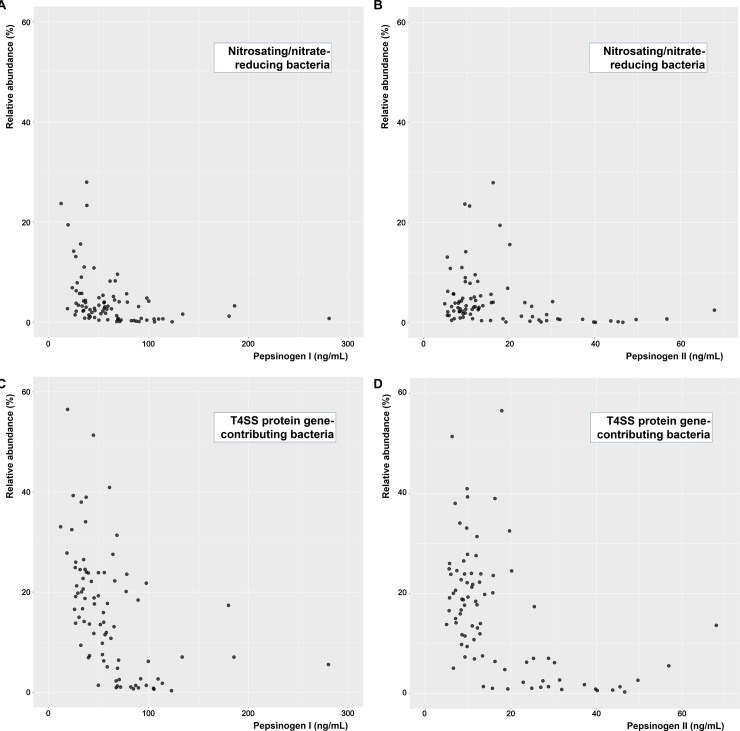
Relationship between bacterial abundance and pepsinogen level. Nitrosating/nitrate-reducing bacteria and (A) PG I or (B) PG II. T4SS protein gene-contributing bacteria and (C) PG I or (D) PG II. PG, pepsinogen; T4SS, type IV secretion system.

**Fig 2:**
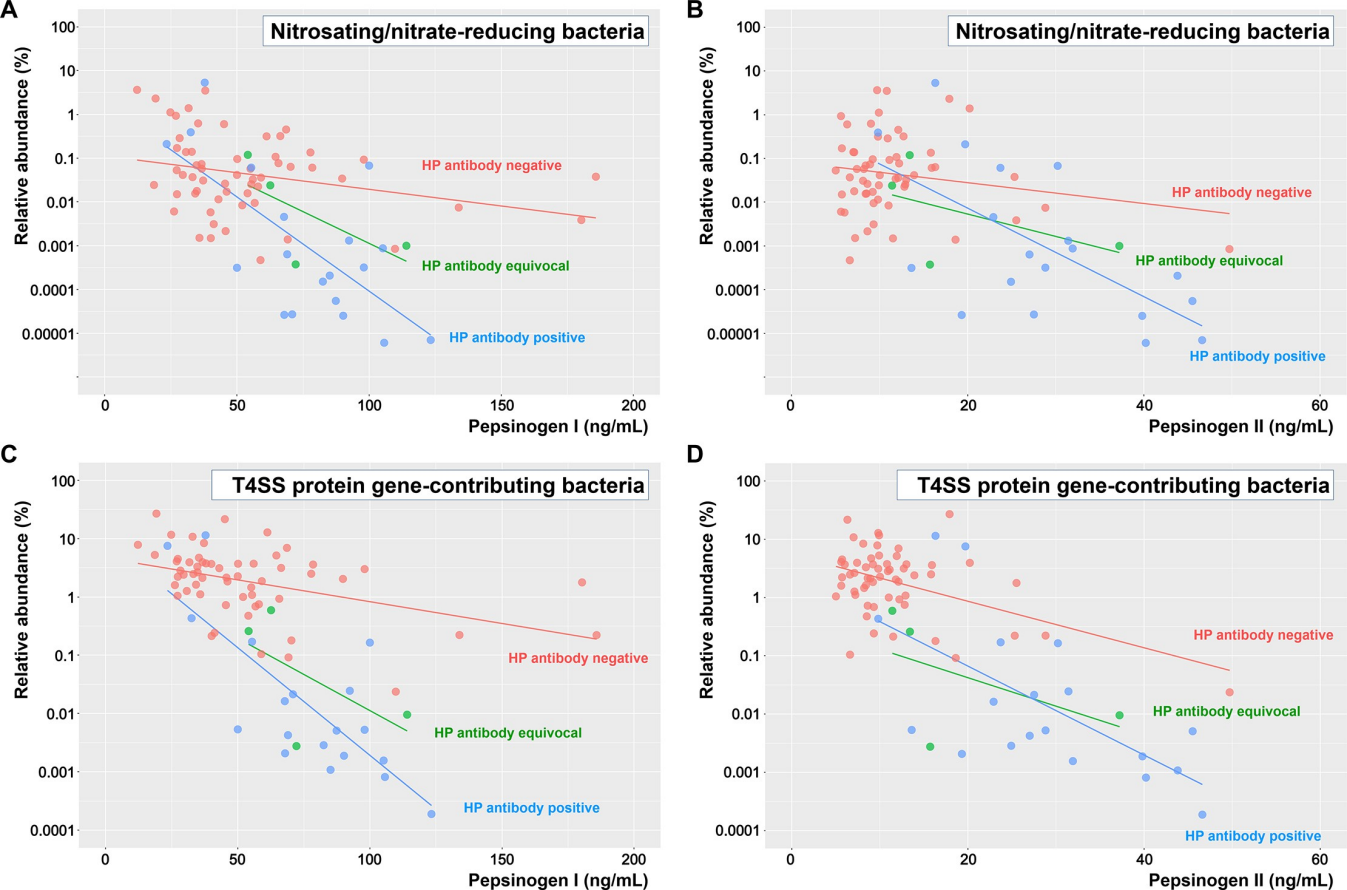
Relationship between bacterial abundance and pepsinogen level according to results of IgG anti-HP antibody testing. Nitrosating/nitrate-reducing bacteria and (A) PG I or (B) PG II. T4SS protein gene-contributing bacteria and (C) PG I or (D) PG II. Blue, green, and red lines indicate regression lines of participants with positive, equivocal, and negative IgG anti-HP antibody results, respectively. Bacterial abundance was expressed on a log scale. PG, pepsinogen; HP, *Helicobacter pylori*; T4SS, type IV secretion system.

**Fig 1** shows the relationship between the relative abundance of bacteria (nitrosating/nitrate-reducing bacteria or T4SS protein gene-contributing bacteria) and PG levels, which seemed to exhibit an exponential inverse correlation. Specifically, the bacterial abundance increased exponentially as PG levels decreased. The relationship between relative abundances and PG levels according to the results of HP infection status are shown in **Fig 2**. Participants with negative IgG anti-HP antibody results had a higher abundance of both nitrosating/nitrate-reducing and T4SS protein gene-contributing bacteria than those with positive results did. Bacterial abundance increased as PG levels decreased in each subgroup according to the results of the IgG anti-HP antibody analysis.

### Predictive model for bacterial abundance

**Table 2** illustrates predictive models for the logarithm of the abundance of nitrosating/nitrate-reducing bacteria. In the multivariable analysis, advanced age, negative IgG anti-HP antibody results, low PG I, low PG II (model 1) or high PG I/II ratio (model 2), and high Charlson comorbidity index were identified as factors associated with the relative abundance of nitrosating/nitrate-reducing bacteria. The adjusted coefficient of determination (R^2^) values of models 1 and 2 were 53.7% and 49.1%, respectively.

**Table 2:** Predictive model for the logarithm of abundance of non-*Helicobacter pylori* nitrosating/nitrate-reducing bacteria.

Variable	Univariable analysis			Multivariable analysis					
	β	SE	*P*-value	Model 1			Model 2		
				β	SE	*P*-value	β	SE	*P*-value
Age, per year	0.019	0.008	0.022	0.018	0.007	0.008	0.015	0.007	0.031
Male	-0.024	0.300	0.937						
IgG anti-*H*. *pylori* antibody									
Negative	0.000			0.000			0.000		
Equivocal	-0.887	0.595	0.140	-0.176	0.484	0.717	-0.339	0.509	0.508
Positive	-1.649	0.304	<0.001	-0.816	0.334	0.017	-1.316	0.334	<0.001
Pepsinogen I, per ng/mL	-0.019	0.004	<0.001	-0.004	0.005	0.439	-0.013	0.004	<0.001
Pepsinogen II, per ng/mL	-0.074	0.011	<0.001	-0.048	0.017	0.007			
Pepsinogen I/II ratio	0.097	0.090	0.281				0.041	0.084	0.629
Body mass index, per kg/m^2^	0.027	0.041	0.511						
Smoking habit									
Never	0.000								
Former	0.539	0.420	0.204						
Current	-0.527	0.395	0.186						
Charlson comorbidity index, per point	1.147	0.302	<0.001	0.745	0.244	0.003	0.777	0.258	0.004
(Intercept)				-3.421	0.325	<0.001	-3.567	0.496	<0.001
Adjusted R^2^, %				53.7			49.1		

T4SS, type 4 secretion system; β, coefficient of regression; SE, standard error; R^2^, coefficient of determination

The predictive model for T4SS gene-contributing bacteria is shown in **Table 3**. The factors associated with T4SS genes-contributing bacteria were similar to those with nitrosating/nitrate-reducing bacteria. In the multivariable analysis, a negative result in the IgG anti-HP antibody test, low PG I, low PG II (model 1) or high PG I/II ratio (model 2), and high Charlson comorbidity index were identified as factors associated with the relative abundance of T4SS protein gene-contributing bacteria. The adjusted R^2^ of models 1 and 2 were 70.0% and 66.7%, respectively.

**Table 3:** Predictive model for the logarithm of abundance of T4SS protein genes-contributing bacteria.

Variable	Univariable analysis			Multivariable analysis					
	β	SE	*P*-value	Model 1			Model 2		
				β	SE	*P*-value	β	SE	*P*-value
Age, per year	0.007	0.008	0.396						
Male	-0.058	0.277	0.836						
IgG anti-*H*. *pylori* antibody									
Negative	0.000			0.000			0.000		
Equivocal	-1.615	0.430	<0.001	-1.139	0.357	0.002	-1.211	0.377	0.002
Positive	-2.099	0.220	<0.001	-1.286	0.247	<0.001	-1.596	0.248	<0.001
Pepsinogen I, per ng/mL	-0.020	0.004	<0.001	-0.005	0.003	0.156	-0.014	0.003	<0.001
Pepsinogen II, per ng/mL	-0.086	0.008	<0.001	-0.041	0.013	0.002			
Pepsinogen I/II ratio	0.228	0.079	0.005				0.087	0.062	0.162
Body mass index, per kg/m^2^	0.023	0.037	0.544						
Smoking habit									
Never	0.000								
Former	0.204	0.396	0.608						
Current	-0.253	0.372	0.499						
Charlson comorbidity index, per point	0.553	0.297	0.066	0.378	0.167	0.026	0.396	0.178	0.029
(Intercept)				-1.055	0.162	<0.001	-1.477	0.295	<0.001
Adjusted R^2^, %				70.0			66.7		

T4SS, type 4 secretion system; β, coefficient of regression; SE, standard error; R^2^, coefficient of determination

The permutational analysis of variance showed that age, PG I, PG II, IgG anti-HP antibody, and Charlson comorbidity index had significant effects on the overall bacterial community (age: R^2^ = 5.0%, *P* < 0.001; PG I: R^2^ = 9.3%, *P* < 0.001; PG II: R^2^ = 10.0%, *P* < 0.001; IgG anti-HP antibody: R^2^ = 16.8%, *P* < 0.001; Charlson comorbidity index: R^2^ = 1.6%, *P* = 0.046).

### Relationship between other bacterial subgroups and serologic testing

To evaluate whether IgG anti-HP antibody and PG levels were associated with bacterial subgroups other than nitrosating/nitrate-reducing bacteria and T4SS protein gene-contributing bacteria, the relative abundance of Cyanobacteria and T3SS protein gene-contributing bacteria was presented according to the IgG anti-HP antibody and PG levels (**S5 and S6 Figs**). There appeared to be an inverse relationship between PG levels and the relative abundance of Cyanobacteria and T3SS protein gene-contributing bacteria, as shown in **S5 Fig**; however, the relative abundance of Cyanobacteria did not significantly correlate with PG I and II (**S6 Fig**). Additionally, no significant association was observed between the relative abundance of T3SS protein gene-contributing bacteria and PG I levels (*P* = 0.469).

## Discussion

HP infection is a well-known risk factor for gastric carcinogenesis [3]. However, various risk factors other than HP infection, including host and environmental factors, have also been suggested [22]. Especially, bacteria other than HP such as nitrosating/nitrate-reducing bacteria may also affect the development of gastric cancer by producing N-nitroso compounds [23]. In addition, interest in T4SS protein gene-contributing bacteria is also increasing because these bacteria are abundant in the stomach of patients with intestinal metaplasia [12]. Because T4SS is an essential protein for transferring cytotoxin-associated gene A (CagA), which is an initial step of gastric carcinogenesis [24], there is a possibility that T4SS protein gene-contributing bacteria may contribute to triggering gastric cancer development. Although nitrosating/nitrate-reducing bacteria and T4SS protein gene-contributing bacteria have not been fully evaluated, we are currently expanding our understanding of these bacteria owing to recent advances in high-throughput sequencing techniques [13]. Determination of the abundance of intragastric bacteria in individuals helps to facilitate the stratification of gastric cancer risks.

However, gastric microbiome analysis requires an upper endoscopy to obtain gastric samples (mucosal tissue or gastric juice). Besides, the interpretation of sequence data obtained from the next-generation sequencing technique is relatively difficult. Therefore, it is not widely used clinically. On the other hand, conventional methods such as rapid urease test or histologic examination can test only the presence of HP among various intragastric bacteria. In our study, we suggested that potential gastric carcinogenesis-associated bacteria may be predicted using serologic testing of IgG anti-HP antibody and PG levels. Although the R^2^ values of the predictive models were not sufficient to accurately predict the bacterial abundance, estimates of bacterial abundance could be used for risk stratification. Because negative results of IgG anti-HP antibody and low PG levels were associated with a high abundance of both nitrosating/nitrate-reducing bacteria and T4SS protein gene-contributing bacteria, patients who showed these results should be made aware of their high risk of gastric cancer. In those patients, negative results of IgG anti-HP antibody results indicate previous infection and spontaneous clearance of HP, rather than an HP infection-naïve status.

In contrast, patients with positive anti-HP antibody results and high PG levels may not need to be overly concerned about gastric cancer because they may have fewer gastric carcinogenesis-related bacteria other than HP. However, over time, chronic gastritis may progress to atrophic gastritis or intestinal metaplasia, and PG levels are expected to decrease gradually. Therefore, periodic serological testing may be needed to assess changes in the gastric environment of these patients.

The relatively high abundance of nitrosating/nitrate-reducing bacteria and T4SS protein gene-contributing bacteria in patients with negative results of anti-HP antibody and high PG levels should be cautiously interpreted. These patients may not have current or past HP infection, and the risk of gastric cancer is extremely low [25], despite their relatively high abundance of nitrosating/nitrate-reducing bacteria and T4SS protein gene-contributing bacteria. Without HP infection, the high abundance of nitrosating/nitrate-reducing bacteria and T4SS protein gene-contributing bacteria alone does not indicate a high risk of gastric cancer.

**Fig 3:**
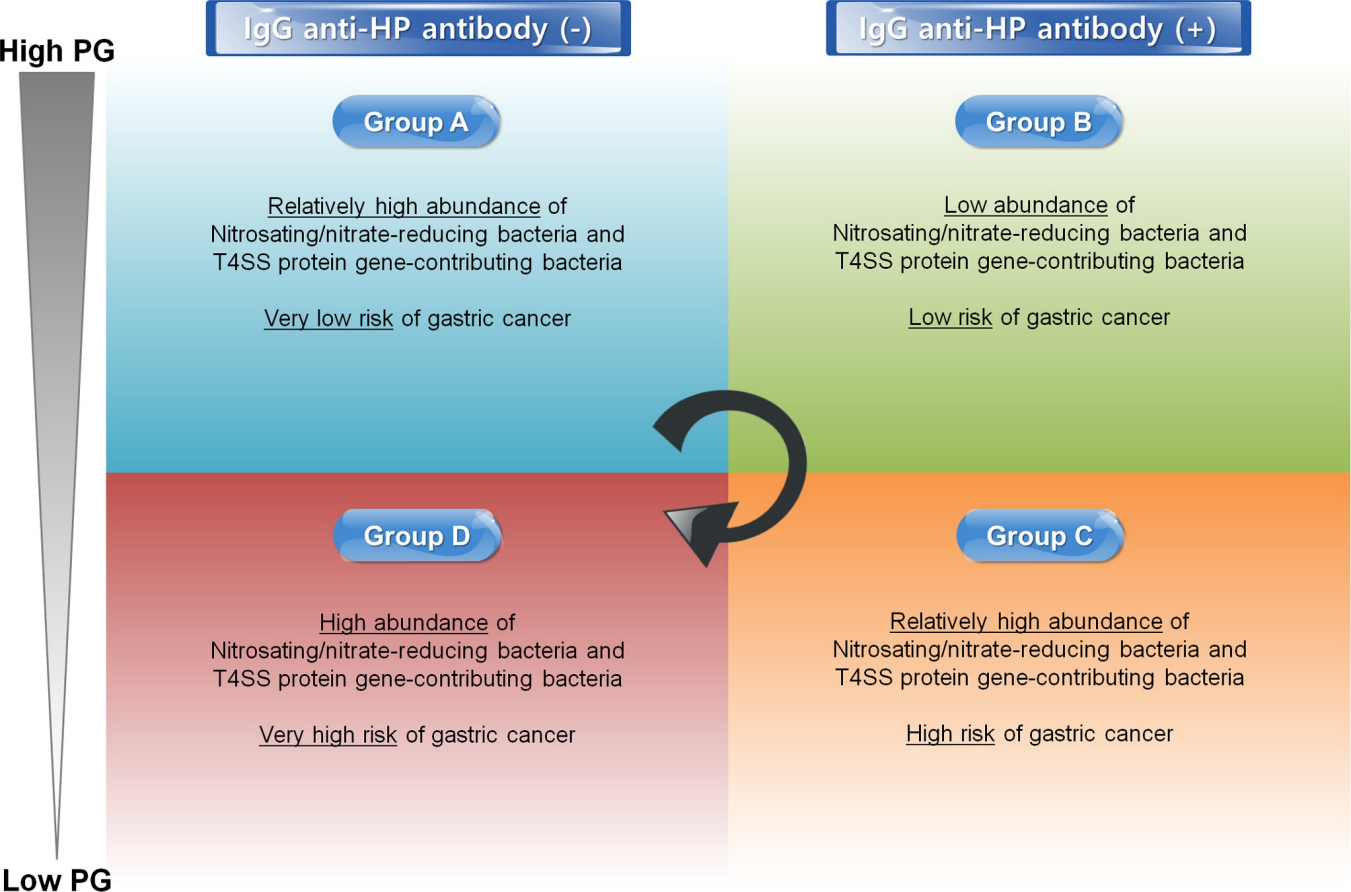
Concept of ABCD method for gastric cancer screening and prediction of gastric microbiome using IgG anti-*Helicobacter pylori* antibody and pepsinogen. HP, *Helicobacter pylori*; PG, pepsinogen.

Prediction of bacterial abundance in the stomach based on serologic testing is similar to the ABCD method, which is a risk stratification method for gastric cancer prediction based on the IgG anti-HP antibody and PG levels (**Fig 3**) [25]. In the ABCD method, patients can be classified into the following four groups: (1) A: IgG anti-HP antibody (-) and high PG levels, (2) B: IgG anti-HP antibody (+) and high PG levels, (3) C: IgG anti-HP antibody (+) and low PG levels, and (4) D: IgG anti-HP antibody (-) and low PG levels. The risk of gastric cancer typically increases from group A to group D. Group A consists of patients who are HP infection-naïve. Groups B and C consist of patients who are HP-infected without and with atrophic gastritis, respectively. Additionally, group D consists of patients with past HP infection. Applying the results of our study to the ABCD method, patients in group D (negative IgG anti-HP antibody results and low PG levels) have a high abundance of nitrosating/nitrate-reducing bacteria and T4SS protein gene-contributing bacteria and, consequently, a very high risk of gastric cancer. In addition, patients in group B (positive IgG anti-HP antibody result and high PG levels) have a low abundance of nitrosating/nitrate-reducing bacteria and T4SS protein gene-contributing bacteria and, therefore, low risk of gastric cancer. In patients in group A (negative IgG anti-HP antibody results and high PG levels), the risk of gastric cancer is extremely low despite a relatively high abundance of nitrosating/nitrate-reducing bacteria and T4SS protein gene-contributing bacteria because they have not current or past HP infection.

In our study, the relative abundance of nitrosating/nitrate-reducing bacteria and T4SS protein gene-contributing bacteria increased as PG levels decreased. Additionally, Cyanobacteria and T3SS protein gene-contributing bacteria tended to be more abundant in patients with low PG levels. Generally, low PG levels are associated with decreased gastric acid secretion and high intragastric pH. Therefore, various bacterial taxa may have the opportunity to increase in patients with low PG levels. However, the relative abundance of Cyanobacteria and T3SS protein gene-contributing bacteria did not significantly correlate with PG levels. In other words, PG levels may be more helpful to predict potential gastric carcinogenesis-related bacteria, such as nitrosating/nitrate-reducing bacteria and T4SS protein gene-contributing bacteria.

Although our study suggests that bacterial abundance in the stomach may be predicted using serologic testing of IgG anti-HP antibody and PG, it has some limitations. First, the R^2^ values of the models were not high enough for the accurate prediction of gastric microbial abundance. Many other factors, including diet, lifestyle, and genetic factors as well as interactions among intragastric microorganisms, have the potential to affect the gastric microbiome composition. To evaluate the exact abundance of the gastric microbiome, gastric microbiome analysis based on next-generation sequencing should be considered. However, we do not think that determining the exact abundance of gastric microbiomes such as nitrosating/nitrate-reducing bacteria is imperative in clinical practice. Just knowing whether their abundance is high or low could help stratify the risk of gastric cancer. Second, our study was performed in a single institute. Although gastric microbiome analysis tools and serologic testing methods could be unified, a generalization of our results to other institutions or countries may be limited. Different prevalence of HP or differences in dietary habits among countries may influence the prediction model.

Despite these limitations, our data enhance the understanding of the relationship between the results of serologic testing of IgG anti-HP antibody and PG and abundance of nitrosating/nitrate-reducing bacteria and T4SS protein gene-contributing bacteria. Not only HP but also low PG levels may be important to predict the abundance of nitrosating/nitrate-reducing bacteria and T4SS protein gene-contributing bacteria.

## Supporting information

S1 Fig Composition of the gastric microbiome according to the HP infection status. For phylum Proteobacteria, non-*Helicobacter* Proteobacteria (Brown) and *Helicobacter* (OliveDrab) are demonstrated separately.
HP, *Helicobacter pylori*
(TIF)

S2 Fig Linear discriminant analysis of the relative abundance of bacteria in the HP-negative and HP-positive groups. For all bacterial taxa evaluated, a significant difference was observed between the groups (*P* < 0.05 by the Kruskal-Wallis test).
HP, *Helicobacter pylori*; LDA, linear discriminant analysis
(TIF)

S3 Fig Relative abundance of nitrosating/nitrate-reducing bacteria and type IV-secretion-system protein gene-contributing bacteria according to HP infection status. HP, *Helicobacter pylori*.
(TIF)

S4 Fig Distribution of serum pepsinogen I (A) and II (B) according to HP infection status. HP, *Helicobacter pylori*; Hollow gray circle indicates outlier.
(TIF)

S5 Fig Relationship between bacterial abundance and pepsinogen levels. Cyanobacteria and (A) PG I or (B) PG II. T3SS protein gene-contributing bacteria and (C) PG I or (D) PG II.
PG, pepsinogen; T3SS, type III secretion system.
(TIF)

S6 Fig Relationship between bacterial abundance and pepsinogen levels according to the results of IgG anti-HP antibody testing. Cyanobacteria and (A) PG I or (B) PG II. T3SS protein gene-contributing bacteria and (C) PG I or (D) PG II.
No statistically significant relationship was observed between the relative abundance of Cyanobacteria and PG I or II. The proportion of Cyanobacteria was 0% in three patients with negative IgG anti-HP antibody results, despite relatively low PG I and II levels. Additionally, the relative abundance of T3SS protein gene-contributing bacteria was not significantly correlated with PG I (*P* = 0.469), although it was associated with PG II (*P* = 0.003).
Blue, green, and red lines indicate regression lines of participants with positive, equivocal, and negative IgG anti-HP antibody results, respectively.
HP, *Helicobacter pylori*; PG, pepsinogen; Bacterial abundance is expressed on a log scale.
(TIF)
