# Supplementary figures and images for: Helicobacter pylori antibody and pepsinogen testing for predicting gastric microbiome abundance
Source: PLoS One. 2019 Dec 4;14(12):e0225961. doi: 10.1371/journal.pone.0225961 (PMC6892531; doi:10.1371/journal.pone.0225961)

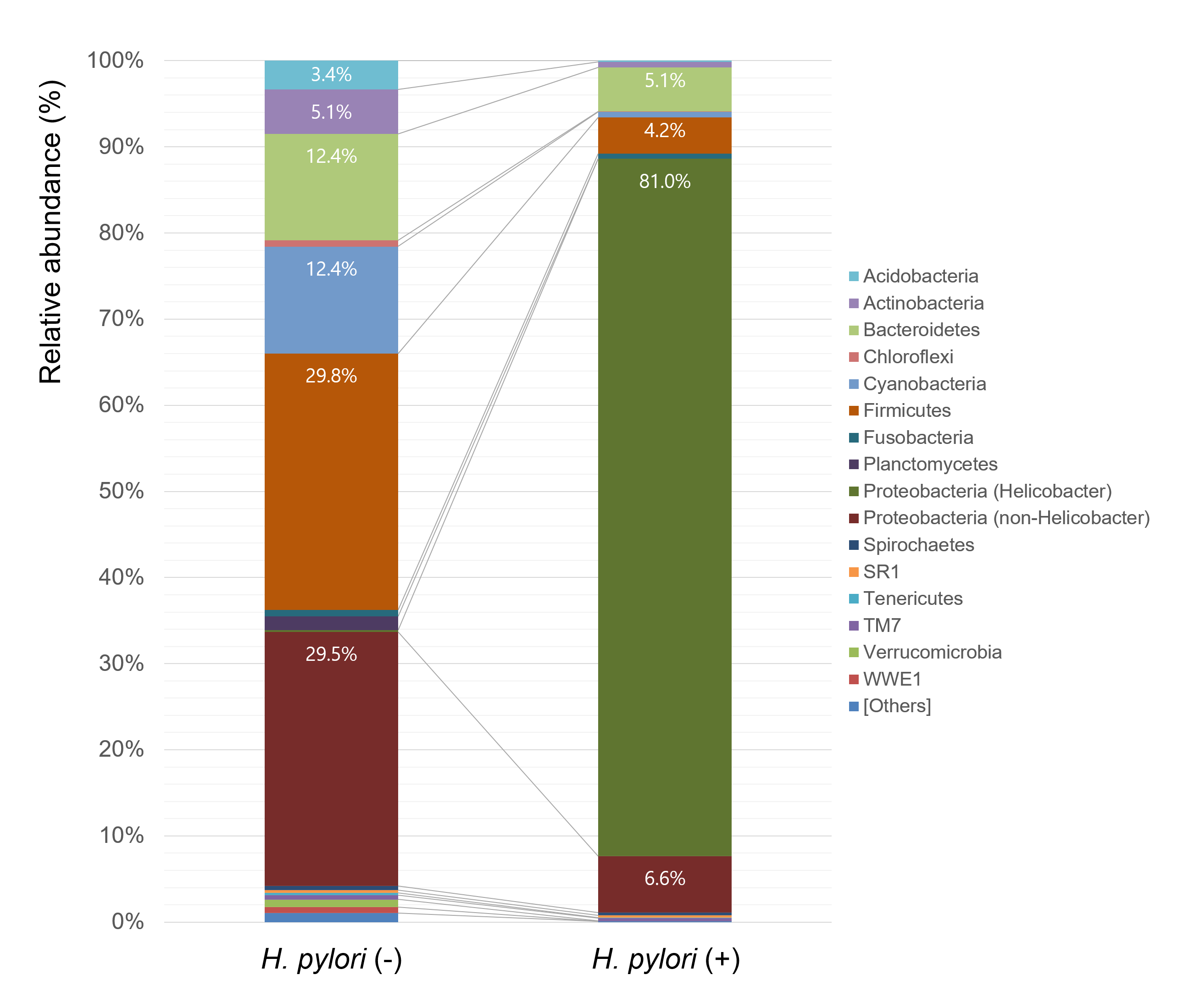

Supplement: S1 Fig — For phylum Proteobacteria, non-Helicobacter Proteobacteria (Brown) and Helicobacter (OliveDrab) are demonstrated separately. HP, Helicobacter pylori (TIF) [file pone.0225961.s001.tif]

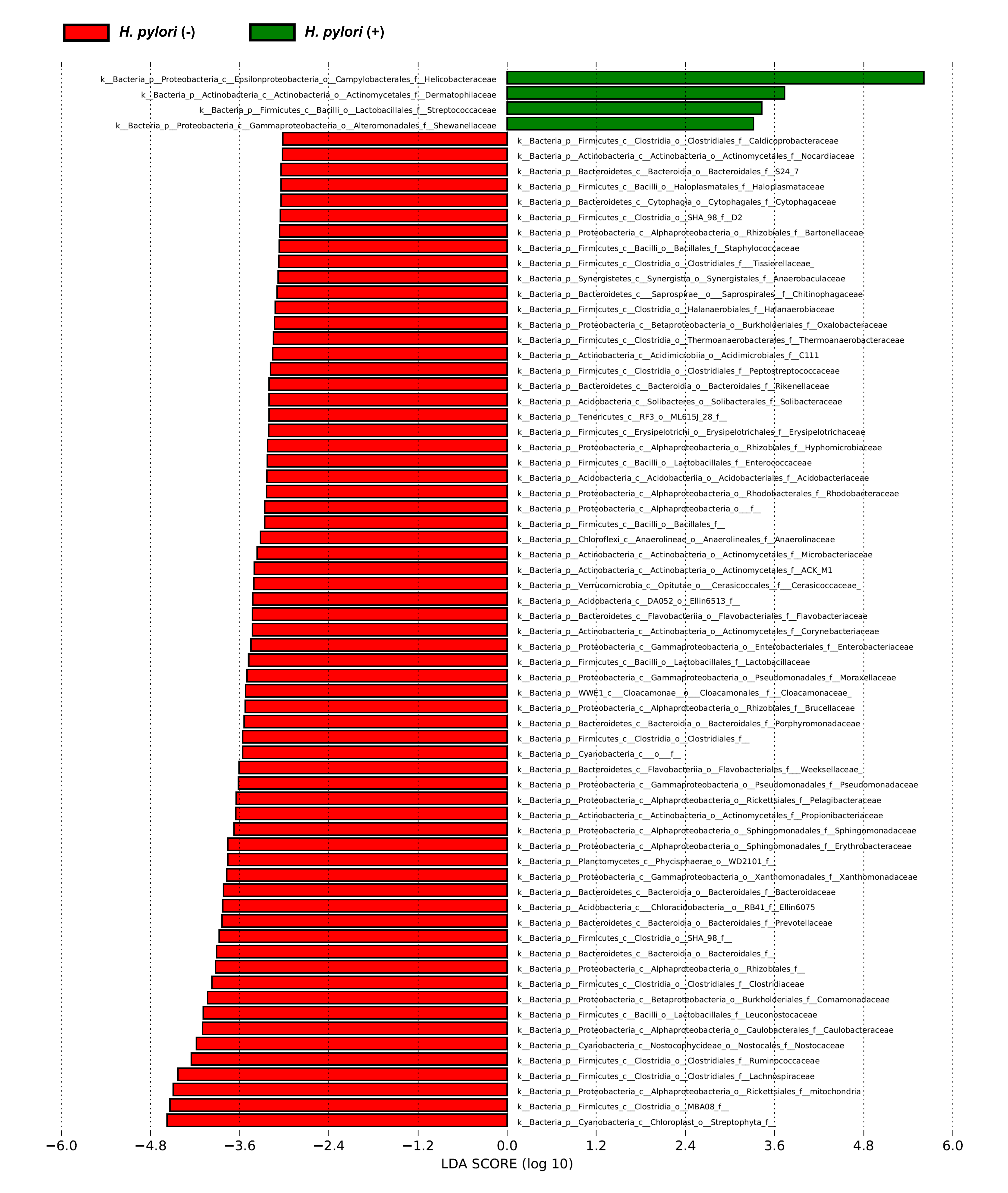

Supplement: S2 Fig — For all bacterial taxa evaluated, a significant difference was observed between the groups (P < 0.05 by the Kruskal-Wallis test). HP, Helicobacter pylori; LDA, linear discriminant analysis (TIF) [file pone.0225961.s002.tif]

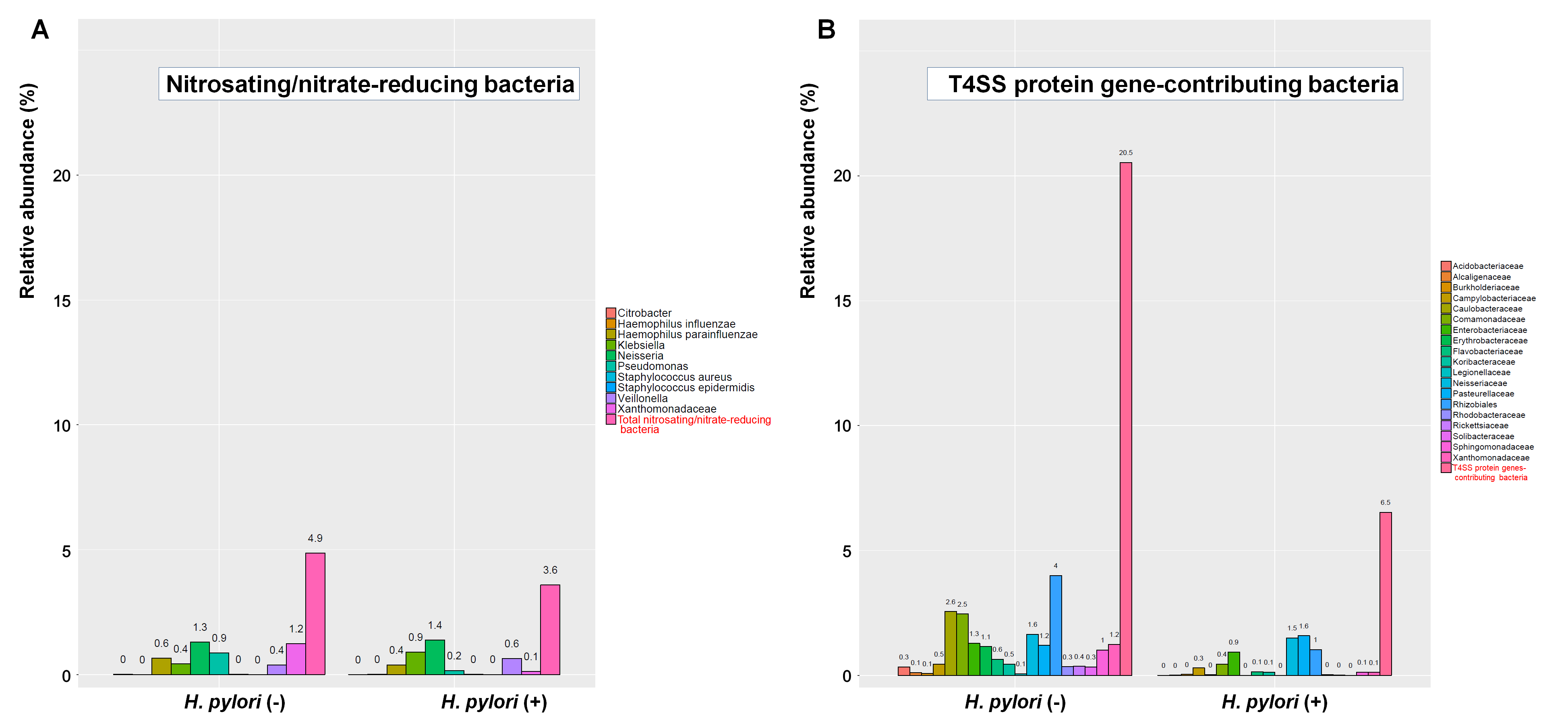

Supplement: S3 Fig — HP, Helicobacter pylori. (TIF) [file pone.0225961.s003.tif]

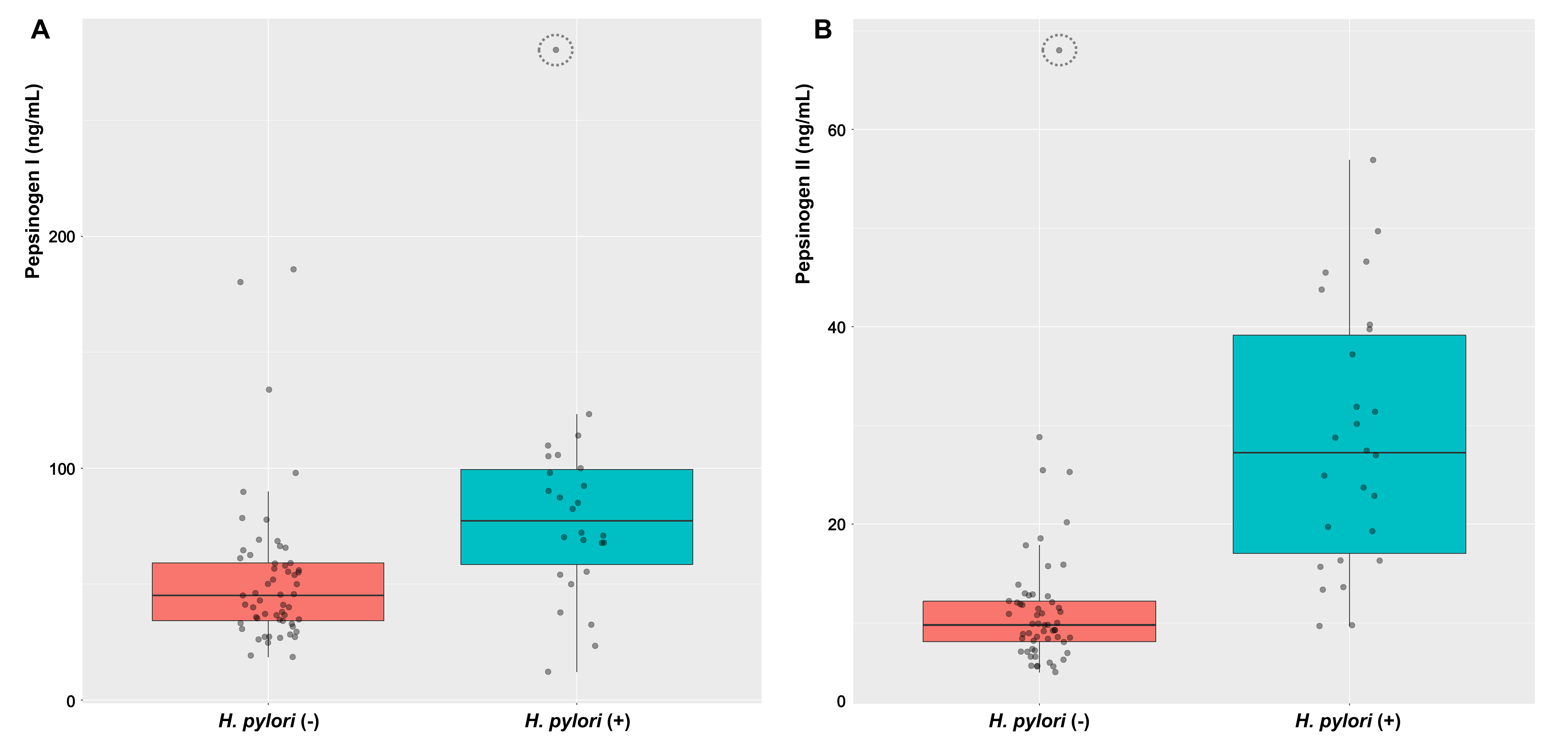

Supplement: S4 Fig — HP, Helicobacter pylori; Hollow gray circle indicates outlier. (TIF) [file pone.0225961.s004.tif]

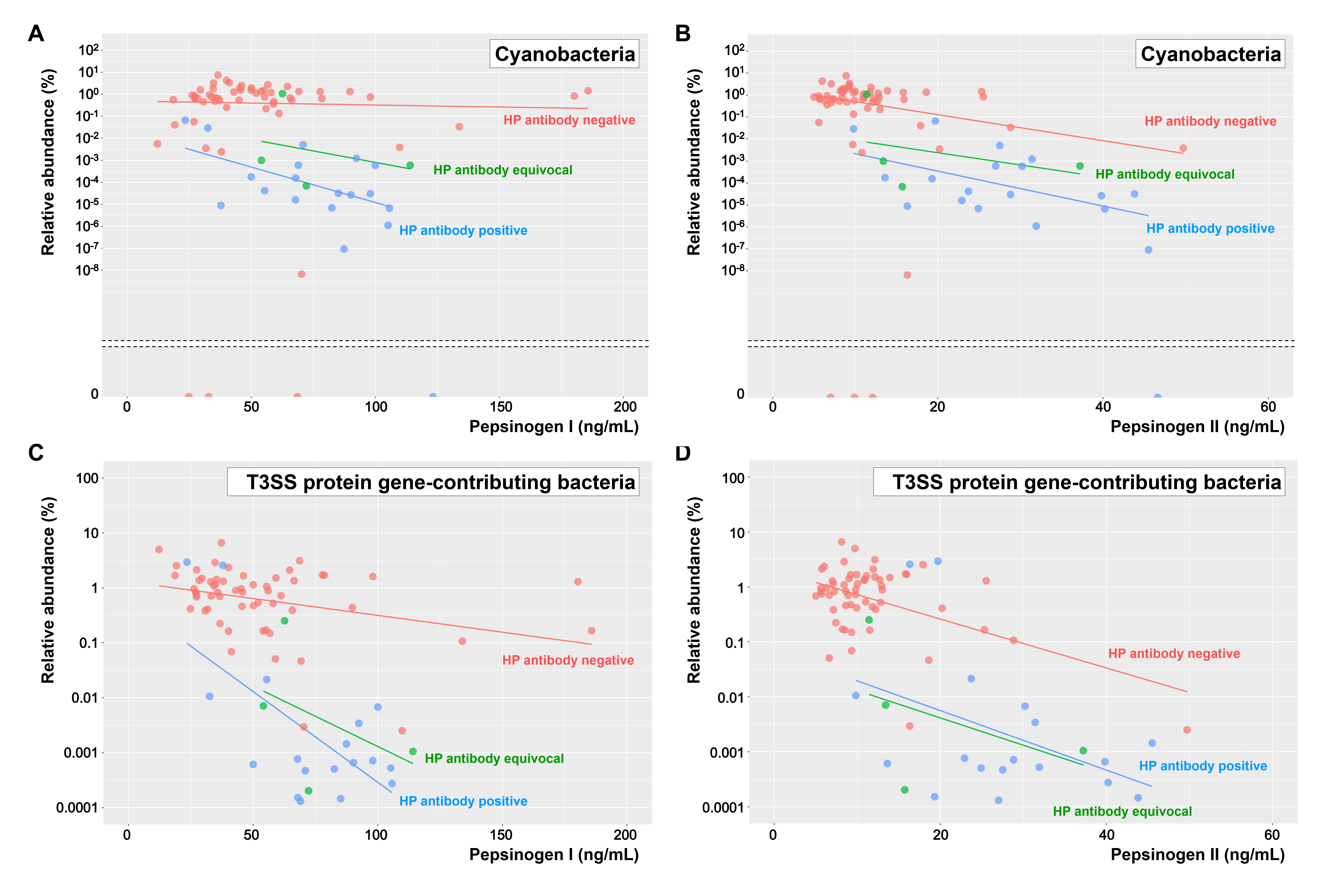

Supplement: S6 Fig — Cyanobacteria and (A) PG I or (B) PG II. T3SS protein gene-contributing bacteria and (C) PG I or (D) PG II. No statistically significant relationship was observed between the relative abundance of Cyanobacteria and PG I or II. The proportion of Cyanobacteria was 0% in three patients with negative IgG anti-HP antibody results, despite relatively low PG I and II levels. Additionally, the relative abundance of T3SS protein gene-contributing bacteria was not significantly correlated with PG I (P = 0.469), although it was associated with PG II (P = 0.003). Blue, green, and red lines indicate regression lines of participants with positive, equivocal, and negative IgG anti-HP antibody results, respectively. HP, Helicobacter pylori; PG, pepsinogen; Bacterial abundance is expressed on a log scale. (TIF) [file pone.0225961.s006.tif]
